# Lsr2, a nucleoid-associated protein influencing mycobacterial cell cycle

**DOI:** 10.1038/s41598-021-82295-0

**Published:** 2021-02-03

**Authors:** Marta Kołodziej, Damian Trojanowski, Katarzyna Bury, Joanna Hołówka, Weronika Matysik, Hanna Kąkolewska, Helge Feddersen, Giacomo Giacomelli, Igor Konieczny, Marc Bramkamp, Jolanta Zakrzewska-Czerwińska

**Affiliations:** 1 Department of Molecular Microbiology, Faculty of Biotechnology, University of Wrocław, Wrocław, Poland; 2 Intercollegiate Faculty of Biotechnology of University of Gdansk and Medical University of Gdansk, University of Gdansk, Gdansk, Poland; 3 Institut Für Allgemeine Mikrobiologie, Christian-Albrechts-Universität Zu Kiel, 24118 Kiel, Germany

**Keywords:** Microbiology, Cellular microbiology

## Abstract

Nucleoid-associated proteins (NAPs) are responsible for maintaining highly organized and yet dynamic chromosome structure in bacteria. The genus *Mycobacterium* possesses a unique set of NAPs, including Lsr2, which is a DNA-bridging protein. Importantly, Lsr2 is essential for the *M. tuberculosis* during infection exhibiting pleiotropic activities including regulation of gene expression (mainly as a repressor). Here, we report that deletion of *lsr2* gene profoundly impacts the cell morphology of *M. smegmatis,* which is a model organism for studying the cell biology of *M. tuberculosis* and other mycobacterial pathogens. Cells lacking Lsr2 are shorter, wider, and more rigid than the wild-type cells. Using time-lapse fluorescent microscopy, we showed that fluorescently tagged Lsr2 forms large and dynamic nucleoprotein complexes, and that the N-terminal oligomerization domain of Lsr2 is indispensable for the formation of nucleoprotein complexes in vivo. Moreover, *lsr2* deletion exerts a significant effect on the replication time and replisome dynamics. Thus, we propose that the Lsr2 nucleoprotein complexes may contribute to maintaining the proper organization of the newly synthesized DNA and therefore influencing mycobacterial cell cycle.

## Introduction

To fit chromosomal DNA into the tiny volume of a bacterial cell, chromosome(s) must be efficiently compacted and organized. Bacterial chromosomes exhibit a hierarchical organization that ranges from large-scale macrodomains (in the Mbp range) to smaller (~ 10 kb) topologically independent microdomains. This hierarchical structure helps maintain both the global chromosome organization and the accessibility of particular chromosomal regions for DNA transactions connected with cellular processes, such as DNA replication, chromosome segregation, and transcription; unlike in eukaryotic organisms, these processes occur simultaneously in bacteria. Bacterial chromosomes are primarily organized by diverse sets of NAPs that bend (HU, IHF, Fis), bridge (H-NS), or wrap (Lrp, Dps) the DNA.

The *Mycobacterium* genus encompasses not only human pathogens that have enormous impact on global health (i.e., *Mycobacterium tuberculosis, M. leprae*), but also the saprophytic species, such as *Mycobacterium smegmatis,* which is a model organism for studies on the cell biology of tubercle bacilli*.* Mycobacteria possess a unique multilayered cell envelope and, in contrast to other rod-shaped bacteria, incorporate peptidoglycan precursors at their cell tips^1–3^, which results in faster elongation of the cell inheriting old pole and asymmetrical cell division. Unlike the situation in other extensively studied bacteria, such as *Escherichia coli*, the chromosome is positioned asymmetrically within the mycobacterial cell throughout the entire cell cycle, and its structure is maintained by a unique set of NAPs^4^. Due to their relative lack of sequence homology to *E. coli* counterparts, most of the mycobacterial NAPs have only recently begun to be characterized^5^. Lsr2, one of the principal mycobacterial NAPs, is believed to function similarly to H-NS: it is a structural homolog of H-NS, preferentially binds AT-rich sequences, and is able to bridge distant DNA fragments or form a rigid nucleoprotein filament in vitro^5–9^.

To date, most of the studies on Lsr2 have been limited to in vitro analysis of Lsr2-DNA interactions. In the present study, we sought to explore the biological role of Lsr2 at the single-cell level. We demonstrate that deletion of the *lsr2* gene has profound impacts on cell morphology resulting in the formation of cells that are shorter and wider than the wild type cells. Time lapse-fluorescent microscopy (TLFM) experiments revealed that Lsr2 forms a highly dynamic nucleoprotein complex(es) and influences both the dynamics of replication machinery and the duration of DNA synthesis. Finally, using TLFM and photoactivated localization microscopy (PALM), we show that the N terminus is indispensable for the formation of the Lsr2-DNA complex.

## Results

### Lack of Lsr2 affects cell and chromosome morphology

Since the in vivo studies on mycobacterial Lsr2 protein has been limited only to bulk-culture observations of the bacterial population and the biological role(s) of this protein is still ambiguous, we decided to examine the influence of Lsr2 on cellular processes at the single-cell level. We constructed an *M. smegmatis* strain with deletion of the *lsr2* gene (*msmeg_6092*; see Fig. S5). Consistent with previous reports on similar strains^8, 10, 11^, the *M. smegmatis* Δ*lsr2* strain (Δ*lsr2*) exhibited changes in colony morphology, biofilm formation, and sliding motility in comparison to the wild-type mc^2^ 155 strain (WT). The strain lacking Lsr2 formed round and smooth colonies on 7H10 agar plates (see Fig. S1A), was unable to form biofilm, and could spread on the surface of solid medium (data not shown).

**Figure 1:**
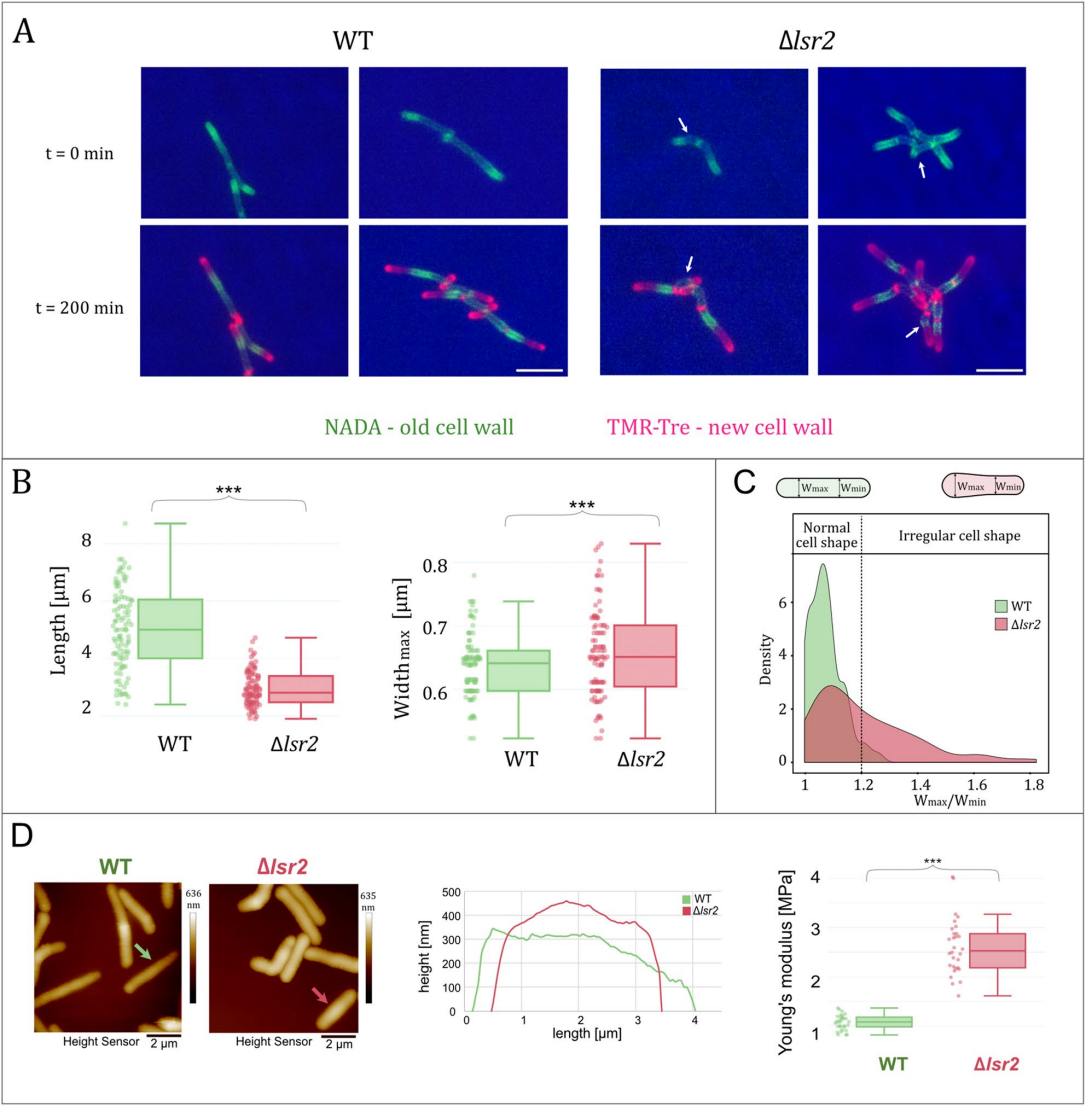
Influence of *lsr2* deletion on mycobacterial cell morphology and elasticity. (**A**) Micrographs showing representative cells of the WT and Δ*lsr2* labeled with NADA and TMR-Tre dyes. White arrows point to morphological disorders of the Δ*lsr2* cells. Bar 5 µm. (**B**) Comparison of the cell length (left) and width (right) between the wild-type *M. smegmatis* mc^2^ 155 (WT) and Δ*lsr2* strains (n = 100). Statistical significance was defined as ***p < 0.0005 (parametric double-sided t test with pooled SD). (**C**) Distribution (shown as the probability density function) of the width ratio (measured between the thickest (W_max_) and the thinnest (W_min_) part of a given cells) in WT and Δ*lsr2* cells (n = 100 each). (**D**) Height sensor images of WT and Δ*lsr2* cells (left panel). The height profiles of representative cells of each strain (middle panel) are indicated by the green and red arrows, respectively. Young's modulus (a measure of elasticity) were compared for WT and Δ*lsr2* cells. Five uniformly spaced points were selected on each bacterial cell to make measurements [n = 30, right panel; statistical significance was defined as ***p < 0.0005 (parametric double-sided t test with pooled SD)].

To analyze cell morphology, we stained Δ*lsr2* and WT cells using either a lipophilic membrane dye (FM 5–95), fluorescent d-alanine (NADA) and/or fluorescent trehalose (TMR-Tre; for details see “Materials and methods”). We found that the cells with *lsr2* deletion were about 42% shorter (2.9 ± 0.6 vs. 5.0 ± 1.4 µm, respectively, n = 100, *p* = 2 × 10^–16^) and 30% wider (0.94 ± 0.9 µm vs. 0.71 ± 0.07 µm, respectively, n = 80, *p* = 2 × 10^–16^) than the WT cells (mean ± standard deviation, n—observation number, *p* value by pairwise t test with pooled SD) (Fig. 1A,B). Interestingly, we observed a subpopulation of irregular, club-shaped Δ*lsr2* cells that resembled corynebacteria in their shape (Fig. 1A,C). The club-shaped Δ*lsr2* cells usually arose from cells that formed a V-shape before undergoing cell division (see Fig. S3A). Surprisingly, we noticed that the number of club-shaped cells was dependent on the type of chamber used for microscopic examinations. We used ibidi dishes or the CellASIC ONIX Microfluidic Platform (B04A plates); the analyzed cells were placed between an agar layer and a polymer coverslip or trapped in the tight microfluidic chamber, respectively. We observed a higher fraction of club-shaped cells in CellASIC plates than in the ibidi dishes (Fig. S3B). To quantitatively analyze this phenomenon, we calculated the ratio of the width of the thickest (W_max_) to the thinnest (W_min_) part of the cells, W_max_/W_min_ (Fig. 1C, Fig. S3B). We found that in CellASIC plates, 40% of Δ*lsr2* cells exhibited W_max_/W_min_ ≥ 1.2 (n = 100), while the use of ibidi plates reduced the fraction of such cells to 30% (n = 100). In the WT strain, regardless of the microscopic chamber used, only 4–5% of cells showed W_max_/W_min_ ≥ 1.2 (n = 100). Since the ceiling height (0.70 µm) of the trap region in the CellASIC microfluidic chamber is less than the average width of the Δ*lsr2* cells (0.94 µm), we speculate that the cells were squeezed in the trap region and were thus more prone to morphological disorders. To further investigate the cell morphology of Δ*lsr2* mutant cells, we performed atomic force microscopy (AFM) analysis. In general, our AFM observations confirmed that Δ*lsr2* cells were shorter and wider than WT cells (Fig. 1D). Since we observed morphological abnormalities of Δ*lsr2* cells, we decided to examine their stiffness. For this purpose, cells were immobilized on a PDMS slide and imaged in the PeakForce QNM mode. We then determined Young’s modulus, which defines the relationship between the stress applied on a material and the resulting strain, and is measured in Pascals (Pa, N m^−2^). The Young’s modulus calculated for Δ*lsr2* cells was higher than the corresponding value for WT cells (2.6 ± 0.6 MPa vs. 1.1 ± 0.2 MPa, respectively, n = 30, p < 2 × 10^–16^) (Fig. 1D), indicating that the mutant cells are more rigid (presumably due to a high turgor pressure, see “Discussion”).

Since the lack of Lsr2 leads to the formation of shorter cells, we sought to determine the elongation rate of cells lacking Lsr2 protein. Using time-lapse microscopy, we compared the cell elongation rate in the Δ*lsr* and WT strains. Our results showed that the lack of Lsr2 resulted in a decreased cell elongation rate (Δ*lsr2* 1.1 ± 0.3 µm × h^−1^ vs. WT 1.5 ± 0.5 µm × h^−1^, n = 50, *p* = 1.7 × 10^–4^; see Fig. S2).

**Figure 2:**
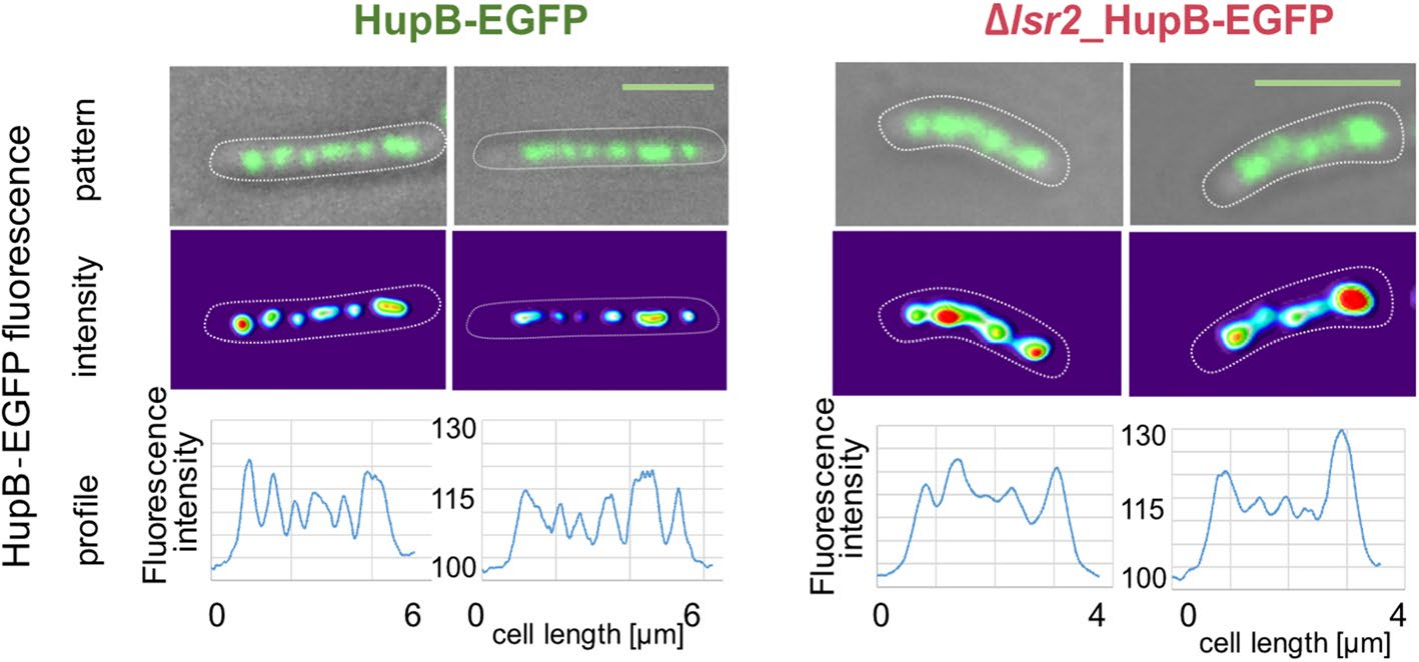
Influence of *lsr2* deletion on global chromosome organization. The nucleoid was visualized using the HupB-EGFP fusion protein. Micrographs and fluorescence intensity profiles of the two representative cells of HupB-EGFP (WT) and Δ*lsr2*_HupB-EGFP strains are shown. Bar 5 µm.

Next, we examined the influence of *lsr2* deletion on chromosome morphology. We used Δ*lsr2* cells that produced HupB fused with enhanced green fluorescent protein (EGFP), which was shown previously to be an efficient chromosome marker^12^. In WT cells, the chromosome was visualized as compact clusters of discrete and bright foci within the cell. Although, the characteristic bead-like structure of the chromosome was preserved in the Δ*lsr2_*HupB-EGFP strain, the HupB-EGFP foci exhibited a more irregular shape; moreover, the foci were closer together in shorter cells, presumably due to the smaller cell volume (Fig. 2). Despite these differences, the deletion of *lsr2* did not affect global chromosome compaction (76 ± 6% of the cell length in WT, n = 440 vs. 80 ± 5% in Δ*lsr2*, n = 444).

Together, these findings indicate that deletion of the *lsr2* gene has a striking effect on single-cell morphology (including changes in cell size) and affects chromosome morphology.

### Deletion of *lsr2* results in a shortened C-period and altered replisome dynamics

**Figure 3:**
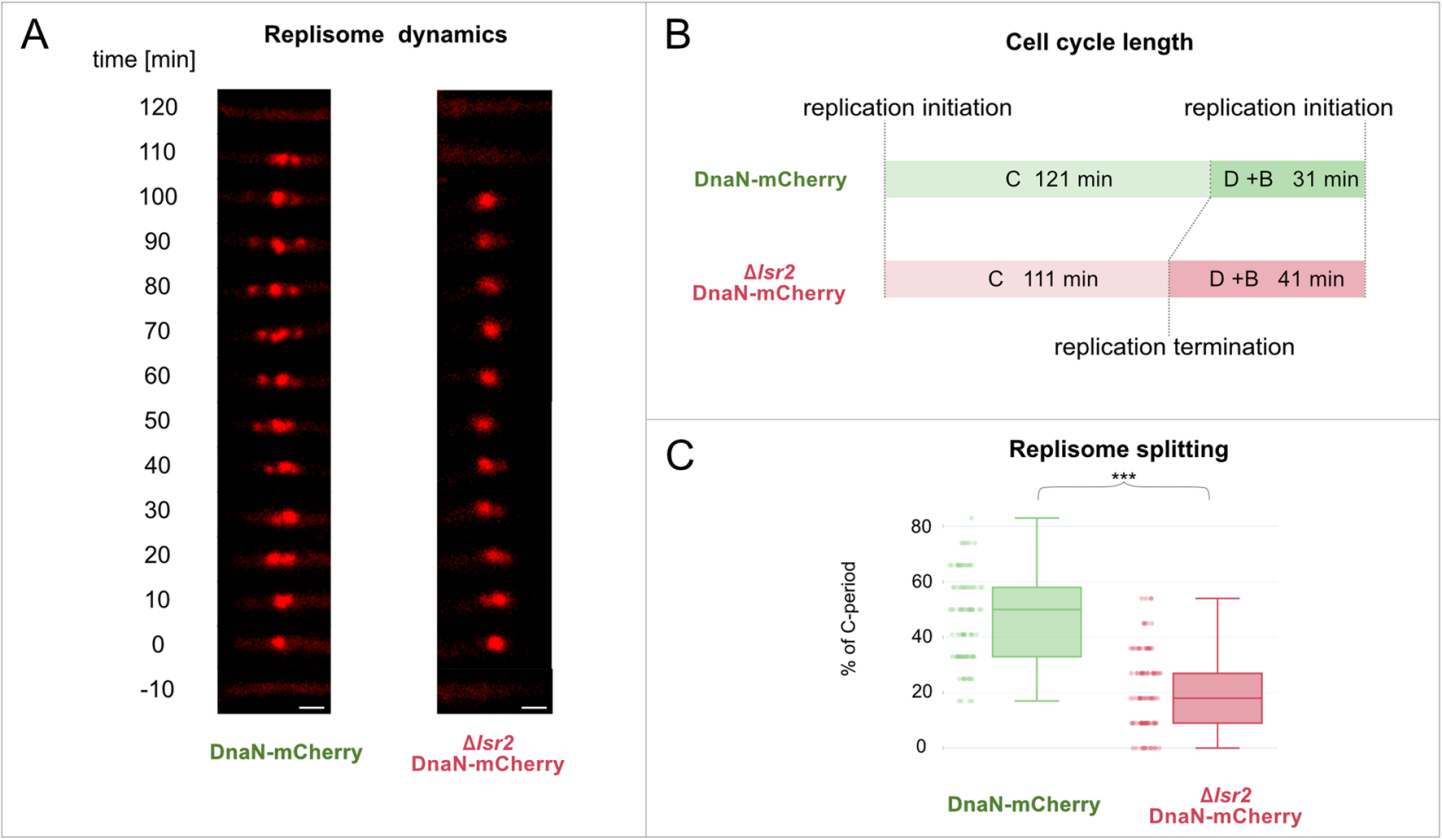
Influence of *lsr2* deletion on the cell cycle and replisome dynamics. (**A**) Real-time microscopic analysis of replisome dynamics during one replication event. In WT cells, replisomes frequently divide and merge; in Δ*lsr2*, in contrast, the replisomes form one fluorescent focus for most of the C-period. Bar 1 µm. (**B**) Schematic representation of the mycobacterial cell cycle in the WT and *lsr2* deletion strains. (**C**) Replisome splitting expressed as the duration when the DnaN-mCherry marked replisomes were observed as more than one fluorescent focus, calculated as a percent of the total replication duration (n = 100, ***p < 0.0005; parametric double-sided t test with pooled SD).

Given that Lsr2 protein was found to have a profound effect on cell and chromosome morphology and cell elongation rate, we sought to elucidate the impact of Lsr2 on the cell cycle. To examine the influence of Lsr2 on cell cycle parameters and the dynamics of chromosome replication (as a key process of the cell cycle), we constructed the Δ*lsr2*_DnaN-mCherry strain (for details see Table S1), which allowed us to perform real-time monitoring of DNA replication in the background of *lsr2* deletion. DnaN, a DNA polymerase III subunit (β-clamp), is frequently fused with fluorescence proteins (FP) to analyze the dynamics of the replisome (the multiprotein complex involved in DNA synthesis). The appearance and disappearance of DnaN-FP fluorescent foci correspond to the initiation and termination of replication, respectively, and the time between these events reflects the duration of replication (the C-period)^13, 14^. The constructed Δ*lsr2*_DnaN-mCherry strain exhibited a colony morphology and growth rate similar to those of the Δ*lsr2* strain (data not shown). TLFM analysis revealed that the duration of chromosome replication in canonically replicating cells (i.e., in cells, in which reinitiation of chromosome replication did not occur) was shorter in the Δ*lsr2_*DnaN-mCherry strain than in the DnaN-mCherry control strain (111 ± 16 min and 121 ± 13 min for Δ*lsr2_*DnaN-mCherry and DnaN-mCherry, respectively, n = 100, *p* = 3.7 × 10^–6^, Fig. 3B), whereas the time between replication termination and replication initiation in daughter cells (the D + B period) was longer (44 ± 19 min and 31 ± 11 min for Δ*lsr2_*DnaN-mCherry and DnaN-mCherry, respectively*,* n = 200, *p* = 6 × 10^–15^). Thus, the overall duration of the cell cycle (B + C + D) remained unchanged (Δ*lsr2*, 152 min vs. DnaN-mCherry control strain, 152 min; see Fig. 3B).

Since deletion of *lsr2* led to shortening of the C-period, we questioned how Lsr2 might influence replisome dynamics during the cell cycle. In *Mycobacterium*, replisomes appear to oscillate around each other, frequently merging and splitting^13^. This dynamics was disturbed in the Δ*lsr2_*DnaN-mCherry strain, as split replisomes were observed in these cells for only 21 ± 14% of the replication time, compared to 47 ± 16% in the DnaN-mCherry strain (n = 100, *p* < 1 × 10^–5^; Fig. 3A,C).

In sum, our TLFM analyses showed that the deletion of *lsr2* gene affects both the duration and the dynamics of DNA replication process.

### Lsr2 exhibits a highly dynamic localization during the cell cycle

**Figure 4:**
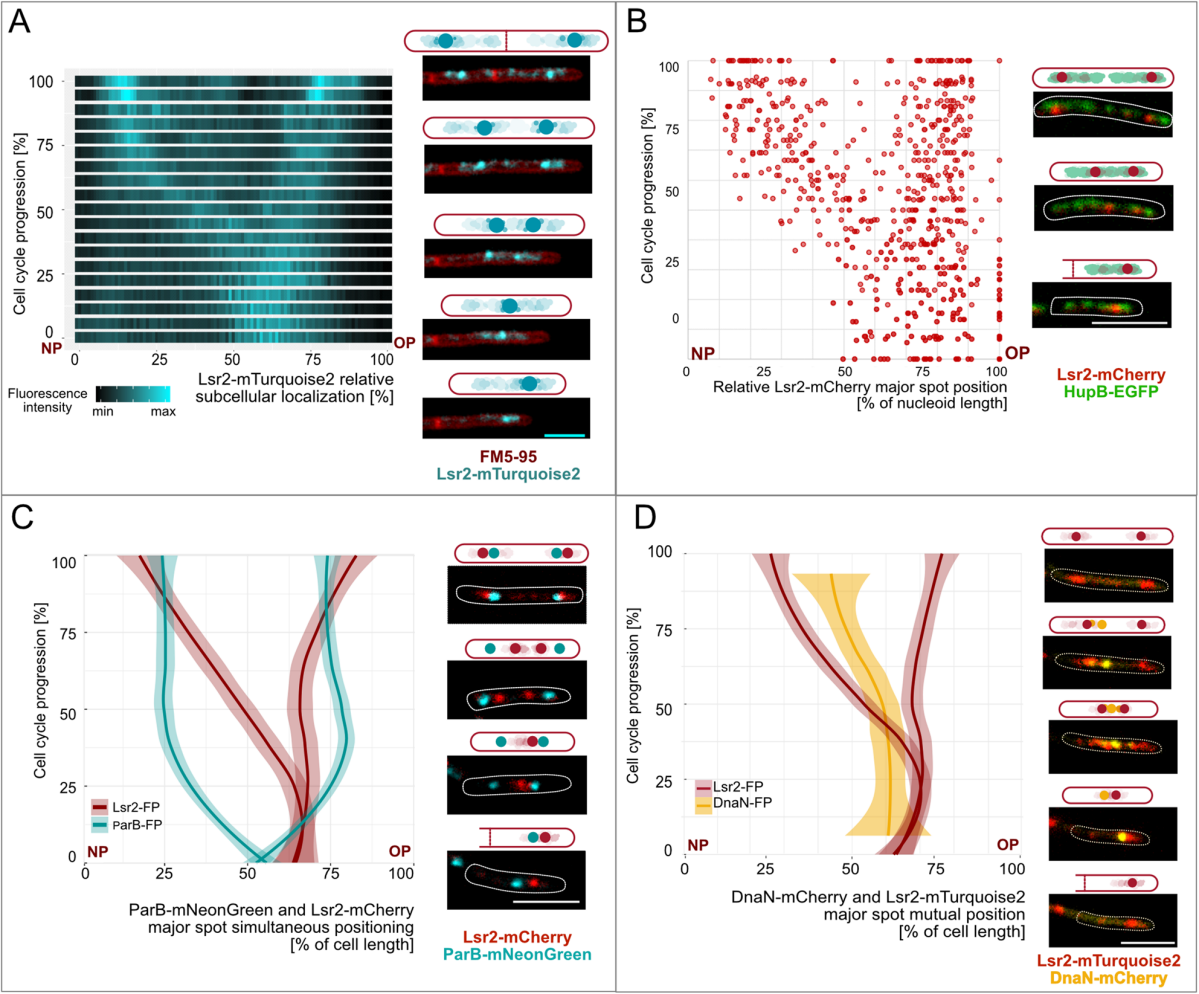
Subcellular localization of Lsr2-FP during the mycobacterial cell cycle. (**A**) Kymograph presenting real-time Lsr2-mTurquoise2 localization (n = 5, left panel) and micrographs of representative FM5-95-stained Lsr2-mTurquoise2 cells. Bar 2 µm (right panel). (**B**) Relative position of Lsr2-mCherry major foci along the nucleoid during the cell cycle (n = 26, left panel) and microscopic images of the Lsr2-mCherry_HupB-EGFP cell confirming that Lsr2-FP localized within the nucleoid area. Bar 5 µm (right panel). (**C**,**D**) Simultaneous positioning of the Lsr2-FP major foci and ParB-mNeonGreen (*oriC*/segrosome) or DnaN-mCherry (replisome) signals over time (left panels). Also shown are graphs representing the averaged position of fluorescent proteins (dark-colored line) and the standard deviation (light-colored ribbon, n = 5). Lsr2-FP and the segrosome(s) localized near one another at the beginning and end of the cell cycle. For most of the C-period, in contrast, the major Lsr2-FP focus (foci) is (are) in close proximity to the replisomes. The images on the right of the sections show time-lapse microscopic images of representative Lsr2-mCherry_ParB-mNeonGreen or Lsr2-mTurquoise2_DnaN-mCherry cells. Bar 5 µm. *NP* new pole, *OP* old pole.

Given that Lsr2 forms extensive oligomers upon DNA binding in vitro^5, 15^ and affects replisome dynamics (as shown immediately above), we decided to analyze its subcellular localization during the cell cycle. For this purpose, we utilized fluorescently-tagged Lsr2 protein. The use of flexible polypeptide linker (see Supplementary Information) between the Lsr2 and the fluorescent protein allowed their independent folding. To avoid potential artifacts in our localization of subcellular Lsr2, we constructed and tested strains that produced Lsr2 fused with different fluorescent proteins (Lsr2-FP, Table 1): EGFP, mCherry, mTurquoise2, and Dendra2 (for details see Table S1). All of them exhibited a colony morphology and growth rate similar to those of the wild-type strain (Fig. S1B), suggesting that the fusion proteins were functioning normally. TLFM revealed that in each analyzed strain, depending on the cell cycle stage, the Lsr2-FP was visible as one or two discrete and bright major foci accompanied by several unstable minor foci (Fig. 4). Additionally, we observed a dispersed fluorescence signal along the cell. The major Lsr2-FP focus (foci) was (were) highly dynamic, frequently changing in size, shape, and intensity (from more compact and dense to ragged and blurred; see Fig. 4A, Video S1). In the case of the Lsr2-EGFP fusion, however, the foci were more clustered and the duplication of the major focus was delayed compared to the other fusions (data not shown). Thus, our observations are consistent with the subcellular localization of H-NS (a structural homolog of Lsr2) fused with GFP. Although GFP (and also EGFP) exhibits very weak dimerization activity, it may act like a “velcro”^16^, and thus could potentially assemble Lsr2-GFP (or H-NS-GFP) into larger complexes. Therefore, in further studies, we decided to use Lsr2 fused with other fluorescent proteins, such as mCherry, mTurquoise2, or Dendra2.

**Table 1:** Fluorescent fusion proteins used in this study (for more details see Table S1).

Fluorescent fusion protein	Application
Lsr2-mCherry Lsr2-mTurquoise2 Lsr2-EGFP	Subcellular localization of Lsr2 in real-time
Lsr2-Dendra2 Lsr2_ΔNTD_-Dendra2	Subcellular localization of Lsr2 in real-time Tracking of Lsr2 single particles in PALM analysis
DnaN-mCherry	Replisome marker
HupB-EGFP	Chromosome marker
ParB-mNeonGreen	*oriC* region marker

To localize Lsr2 along the chromosome, we constructed a strain producing HupB-EGFP (as a chromosome marker) and Lsr2-mCherry fusion proteins from their native chromosomal loci (for details see Table S1). TLFM analysis of the HupB-EGFP_Lsr2-mCherry cells showed that during the entire cell cycle, the major Lsr2 focus (or two foci) and the weaker diffused foci were localized only within the area occupied by the nucleoid (Fig. 4B, Video S1). The major focus split into two foci at approximately the 1/3 point of cell cycle progression, and thereafter both of the duplicated foci moved towards opposite cell poles (Fig. 4A). Interestingly, the focus closest to the old cell pole was positioned at a relatively constant distance from the edge of the chromosome (20 ± 10% of the chromosome length, n = 390) at all-time points, while the second one traversed the chromosome to reach the opposite edge (Fig. 4B).

Since the subcellular localization (and dynamics) of the two major Lsr2-mCherry foci during the cell cycle resemble the positioning of segrosomes (i.e., ParB-FP complexes^17^), we decided to track both proteins in the ParB-mNeonGreen_Lsr2-mCherry strain (for details see, Table 1, Table S1) and analyze their dynamics at different time intervals. TLFM observations revealed that at the beginning of the cell cycle, the Lsr2-mCherry and ParB-mNeonGreen foci were positioned slightly off-center, closer to the old cell pole, and they were relatively close together (Fig. 4C). Immediately following the initiation of replication, the ParB-mNeonGreen complex was duplicated and both foci were rapidly segregated towards the opposite cell poles in an asymmetrical manner relative to midcell (Fig. 4C and Ginda et al.^17^). In contrast, the Lsr2-mCherry focus remained around the midcell (for 53 ± 9 min (n = 50) after the ParB-mNeonGreen duplication). This was followed by duplication of the major Lsr2-mCherry focus and the subsequent movement of these foci to the opposite cell poles in asymmetric fashion resembling mode of action of segregation machinery^17^. Shortly before the division of the cell, the translocated Lsr2-mCherry foci exhibited polar localizations that were more pronounced than those of the ParB foci (Fig. 4C, Video S1). Thus, fluorescent foci derived from Lsr2-mCherry and ParB-mNeonGreen were localized close together only at the beginning and at the end of the cell cycle, when chromosome replication did not occur (Figs. 4C, 6).

Because the lack of Lsr2 protein altered the C-period duration and replisome dynamics, we decided to explore the localization of Lsr2-FP in the background of chromosome replication. For this purpose, we constructed a strain that simultaneously produced Lsr2-mTurquoise2 and DnaN-mCherry (for details see Table 1, Table S1). Previous reports showed that *M. smegmatis* replisomes localize near the midcell and are placed asymmetrically on the chromosome^13, 18^. At the time of replication initiation, the replisomes are assembled close to the edge of the chromosome in the old-pole-proximal cell half; before the termination of replication, the replisomes migrate toward the new-pole-proximal cell half. TLFM analysis of the Lsr2-mTurquoise2_DnaN-mCherry strain showed that both proteins localized close to one another for approximately the first half of the C-period. After the duplication of Lsr2-mTurquoise2, replisomes were observed in the vicinity of the new-pole-proximal major focus of Lsr2-mTurquoise (Fig. 4D, Video S1).

Together, these findings reveal that the major Lsr2-FP and ParB-FP complexes are localized relatively close together at the beginning and end of the cell cycle, and the main Lsr2-FP foci are in close proximity to the replisomes (but they do not colocalize) for most of the C-period.

### The oligomerization domain critically influences the subcellular localization of Lsr2

Since Lsr2-FP forms mainly one or two discrete bright foci and binds to hundreds of sites in vivo (as demonstrated ChIP-on-chip experiments^19^), we expected that Lsr2 might use its N-terminal oligomerization domain to bridge/link distal sites on the chromosome and thereby form higher-order nucleoprotein complexes^5^. To test this hypothesis, we constructed strains producing either Lsr2-Dendra2 or its truncated form lacking the N-terminal oligomerization domain (Lsr2_ΔNTD_-Dendra2) (for details including the precise border between both domains see Table S1, Figs. S4, 5B) from the native chromosomal locus. The phenotype of the Lsr2_ΔNTD_-Dendra2 strain resembled that of the Δ*lsr2* strain: it formed round and smooth colonies on 7H10 agar plates (Fig. S1B).

**Figure 5:**
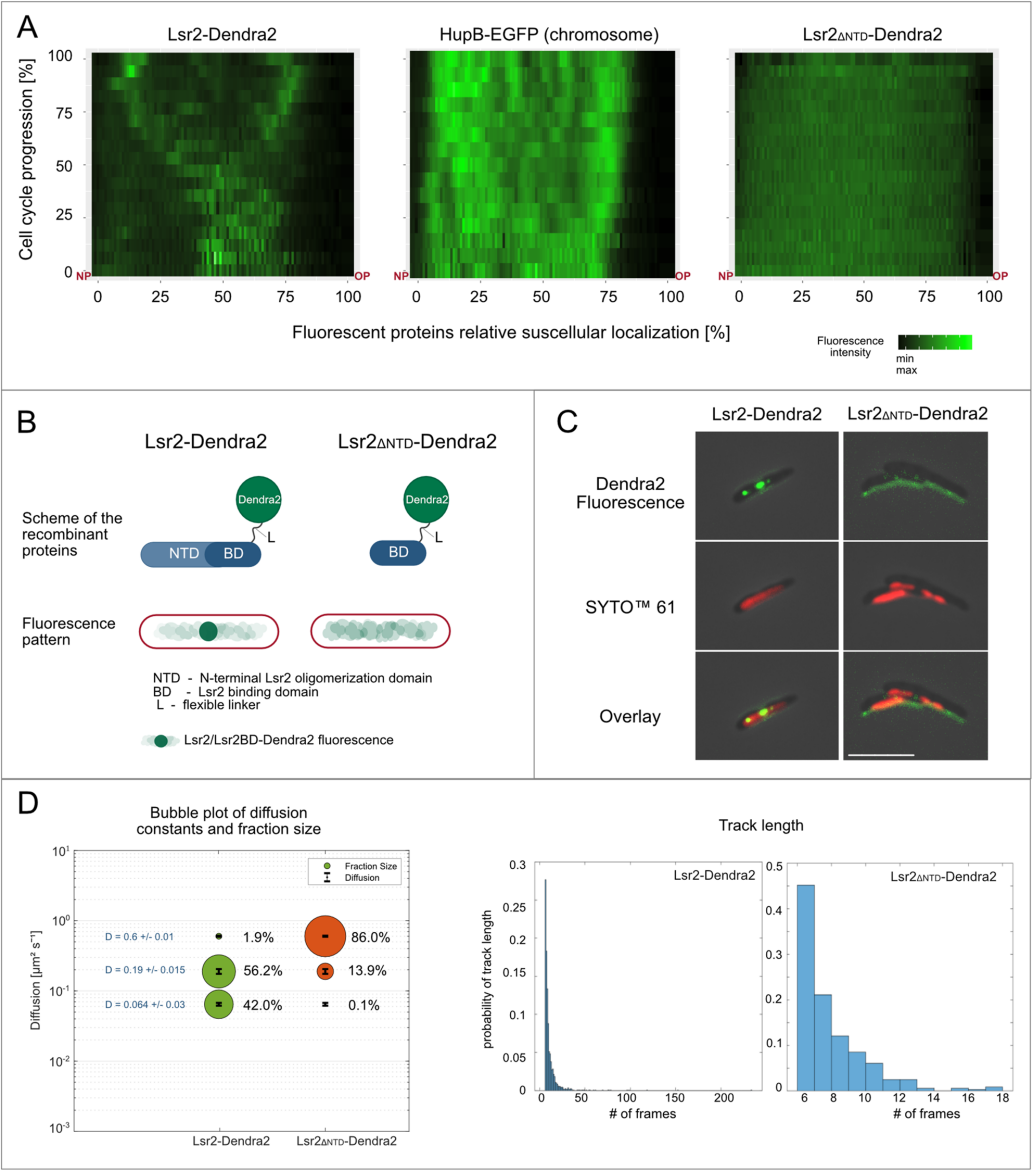
The influence of the N-terminal oligomerization domain on the subcellular localization and DNA binding of Lsr2. (**A**) Fluorescence profiles of Lsr2_ΔNDT_-Dendra2, HupB-EGFP (chromosome), and Lsr2-Dendra2 during the cell cycle (n = 5). The truncated version of Lsr2 is observed as diffused fluorescence along the cell. *NP* new pole, *OP* old pole. (**B**) Graphic representation of full-length and truncated Lsr2 fused with Dendra2 (top panel). Fluorescence patterns of both proteins in *M. smegmatis* cells (bottom panel). (**C**) Microscopic images of representative Lsr2-Dendra2 and Lsr2_ΔNDT_-Dendra2 cells stained with the nucleic acid dye, SYTO™ 61. Bar 5 µm. (**D**) PALM analysis of the mobilities of Lsr2-Dendra2 and Lsr2_ΔNDT_-Dendra2 particles. The bubble plot comparison of diffusion constants and fraction sizes shows that the ratio of diffusing versus immobile particles was higher for the truncated form than for the full-length protein (left panel). The histograms represent the track length of full-length and truncated Lsr2-Dendra2.

Microscopic analysis revealed that, unlike Lsr2-Dendra2, Lsr2_ΔNTD_-Dendra2 was visible as a diffused fluorescence signal spread along the whole cell (Fig. 5A,C). We did not observe any individual Lsr2_ΔNTD_-Dendra2 focus at any point during the cell cycle. This suggests that the N-terminal domain of Lsr2 is indispensable for the formation of nucleoprotein complexes in vivo*.* Moreover, since a substantial fraction of Lsr2_ΔNTD_-Dendra2 was presumably not associated with the chromosome (see Fig. 5A,C, compare HupB-EGFP and Lsr2_ΔNTD_-Dendra2), we postulate that the lack of the N-terminus may also reduce the DNA-binding affinity of the truncated Lsr2 protein in vivo. To verify our hypothesis, we used photoactivated localization microscopy (PALM) to visualize single molecules of Lsr2-Dendra2 and Lsr2_ΔNTD_-Dendra2 and analyze their mobility. Our results showed that the particles of both proteins were heterogeneous in their mobility. For full-length Lsr2-Dendra2 protein, we observed mainly two subpopulations: static molecules (diffusion constant, D = 0.064 ± 0.003 µm^2^ s^−1^) and slow-mobility molecules (D = 0.190 ± 0.015 µm^2^ s^−1^), which represented 42% and 56% of the population, respectively (2060 tracks from 91 cells were analyzed). Only a marginal fraction of the tracks (< 2%) showed high mobility (D = 0.60 ± 0.01 µm^2^ s^−1^). In contrast, 86% (n = 365 tracks obtained from 92 cells) of the truncated protein molecules (Lsr2_ΔNTD_-Dendra2) exhibited high mobility, and only approximately 14% exhibited slow mobility (with D values corresponding to that of full-length Lsr2-Dendra2, Fig. 5D). Notably, we collected significantly fewer tracks for the truncated Lsr2, mainly because there were fewer points per track compared to those of the full-length protein (only tracks consisting of at least five points were included in the analysis). We observed the same tendency regardless of the exposure time applied during data acquisition. Analysis of dwell times (the period during which a particle resides inside a defined radius, which in our case corresponded to the time when Lsr2-Dendra2 or its truncated form remained bound to the chromosome) via SMTracker showed that all molecules of a given protein (fast vs. slow diffusing) exhibited the same dwell times. However, the dwell times calculated for Lsr2_ΔNTD_-Dendra2 were twofold lower than those obtained for full-length Lsr2-Dendra2 (47 ± 4 ms vs. 82 ± 2 ms, respectively).

In sum, our TLFM and PALM analyses demonstrated that the N-terminal domain of Lsr2 is essential for the ability of Lsr2 to form large nucleoprotein complexes in vivo.

## Discussion

The Lsr2 protein plays both architectural and regulatory roles and is highly conserved throughout the Actinobacteria, including *Corynebacterium*^20, 21^, the pathogenic *Mycobacterium,* and the antibiotic-producing *Streptomyces*^22^. Notably, Lsr2 is considered to be essential in *M. tuberculosis*. It regulates the transcription of multiple genes including those involved in the pathogenicity of tubercle bacilli^23, 24^. However, there were no previously published studies regarding the impact of Lsr2 on the chromosome organization and cell cycle in mycobacteria or any other Actinobacteria. We herein used a suite of complementary microscopic techniques (i.e., TLFM, AFM, PALM) to elucidate the influence of Lsr2 on single-cell morphology and the cell cycle of *M. smegmatis,* which is a model organism to study the biology of pathogenic mycobacteria including *M. tuberculosis*.

Our single-cell microscopic observations revealed that mycobacterial cells lacking the Lsr2 protein exhibit morphological abnormalities: shortening and widening of the cell shape, and (in some cases) an irregular club-shaped morphology (Fig. 1, Fig. S3). We speculate that the shortening of the *Δlsr2* cell length is solely due to their reduced cell elongation rate (Fig. S2) and we expect that this phenomenon has influence on other cellular processes. For example, turgor pressure is likely to be increased (due to higher macromolecular crowding) within the shorter cells^25^. Our AFM data supported this assumption: Young’s modulus, which reflects the turgor pressure (related to cell stiffness) was higher for Δ*lsr2* versus WT cells (Fig. 1D). Since the cells lacking Lsr2 exhibit higher turgor pressure and Lsr2 presumably regulates genes that are involved in multiple cellular processes including cell envelope synthesis^15^, we speculate that Δ*lsr2* cells might be more prone to morphological disorders under mechanical tension (e.g., squeezing, which appears also in the ONIX chamber) or/and within the less dense area of cell envelope (i.e., at the snapping site or at the newly formed poles during septum formation and cell division; Fig. 1B). Indeed, we observed a relatively large fraction of club-shaped cells of Δ*lsr2* strain (30–40%, Fig. 1C, Fig. S3B).

As in the case of *h-ns* gene deletion^26^, we did not observe global chromosome decondensation in cells lacking the Lsr2 protein. However, additional factors, such as molecular crowding and depletion forces^27–29^, may also play crucial role in determining the global chromosome architecture. A high turgor pressure is frequently associated with increase in cytoplasmic concentration, macromolecular crowding, and depletion forces^27, 28^. Thus, since the concentration of crowders (e.g., ribosomes) is expected to be higher in smaller cells, the fraction of the cell occupied by the chromosome might be even slightly lower in Δ*lsr2* cells (Fig. 2). Moreover, it should be noted that mycobacteria possess a unique set of NAPs (including Lsr2 and HupB) and a chromosomal architecture that is presumably different than that of *E. coli*.

However, our microscopic observations suggest that the lack of Lsr2 protein causes local changes in the chromosome structure; the HupB-EGFP foci were more irregularly shaped in Δ*lsr2* cells than in the wild-type strain (Fig. 2). Recent comprehensive in vitro studies revealed that HupB interacts with Lsr2, forming a novel DNA-binding entity^30^. Thus, the observed local abnormalities in chromosome organization of Δ*lsr2* cells support previous hypothesis that HupB-Lsr2 interactions may contribute to shaping local chromosome architecture and dynamics^30^. Further application of super-resolution microscopy should shed light on the impact of Lsr2 on the local structure of the chromosome.

**Figure 6:**
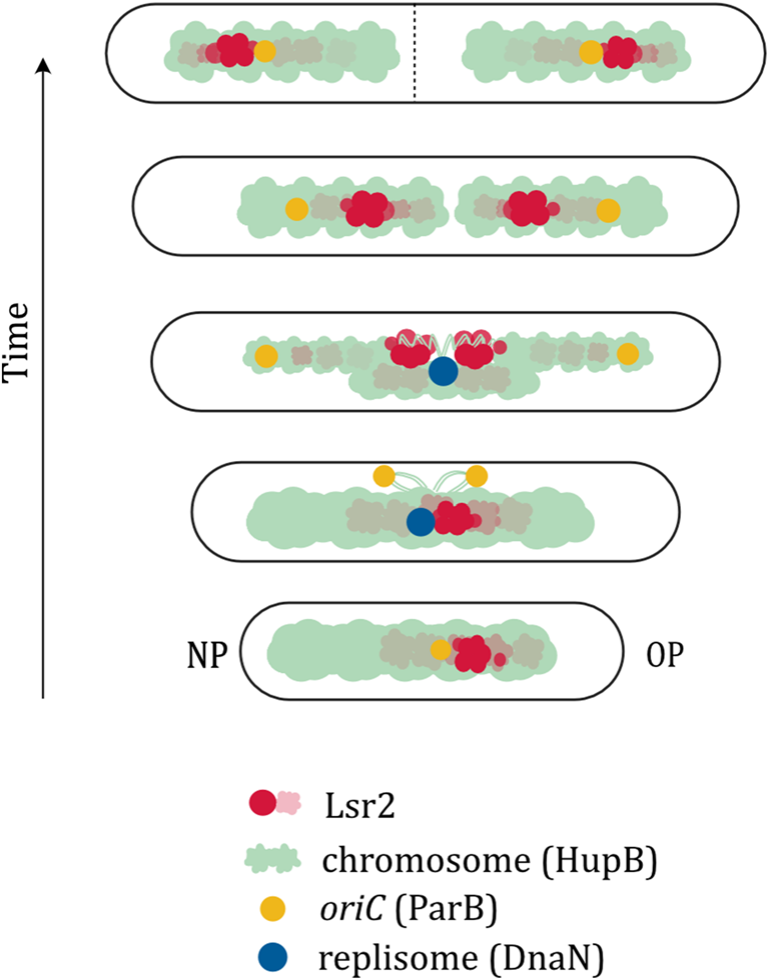
Cartoon illustrating the subcellular localization of Lsr2 during the mycobacterial cell cycle. Lsr2 forms one or two major nucleoprotein complexes. At the beginning of the cell cycle, the single Lsr2 complex is localized slightly asymmetric in relation to the midcell near the *oriC* region. Soon after the initiation of DNA replication, the newly replicated *oriC* regions are rapidly segregated towards the opposite cell poles, while Lsr2 remains localized in the vicinity of replisomes. Next, major Lsr2 complex splits into complexes, which move towards the opposite cell poles where the segrosomes are already located. The cartoon is based on the results obtained from our TLFM (Fig. 4) and PALM (Fig. 5) experiments.

While the biochemistry of the Lsr2 protein has been well studied^5–7, 9^, its subcellular localization and influence on the mycobacterial cell cycle were previously unknown. Our real-time imaging analysis revealed that the fluorescently tagged Lsr2 (Fig. 4A) formed one or two bright major foci, along with several minor complexes and dispersed fluorescence that presumably reflect binding to the single cluster of Lsr2 target sequences and disassembly of nucleoprotein complexes during ongoing replication, respectively. Since Lsr2 was observed in vivo as a large focus/foci within the area occupied by the chromosome (Figs. 4B, 5A,C), we assume that this protein forms nucleoprotein complexes in mycobacterial cells. Their localization is dynamic during the cell cycle. At the beginning and end of the cell cycle, the Lsr2 foci are localized at cell poles near the segrosomes (Figs. 4C, 6, Video S1). For most of the C-period, in contrast, the Lsr2 foci occupy midcell positions near the replisomes. The main Lsr2 focus splits into two foci when approximately half of the chromosome has been copied (see Figs. 4D, 6). Interestingly, our results revealed that the two major Lsr2 foci move asymmetrically—with one traveling a greater distance (this one moving to the new pole). This asymmetric translocation mode of the Lsr2 foci presumably reflects asymmetric action of the segregation machinery^17, 18^ and the unequal division and growth of mycobacterial cells.

Since the major focus (or foci) of Lsr2 is (are) localized in close proximity to the replisomes (Fig. 4D), we speculate that this protein may influence chromosome replication. Indeed, the duration of DNA replication is shorter in Δ*lsr2* cells (Fig. 3B), presumably due to the lack of spatial obstacles (i.e., Lsr2 nucleoprotein complexes). In the WT strain, such obstacles might need to be removed from the DNA ahead of the replication machinery. Notably, the genes involved in the DNA replication are presumably not regulated by Lsr2 as indicated global transcription analysis of *M. smegmatis* Δ*lsr2* strain (performed using DNA microarrays^15^). The presence of Lsr2 nucleoprotein complexes in the vicinity of replisomes may also suggest that, similar to HN-S in *E. coli*^31^, the Lsr2 protein is involved in organizing the sister DNA stretches that are moved away from the replication machinery. Moreover, at around the halfway point of chromosome replication, the DNA must undergo significant conformational changes due to reorganization of the Lsr2 nucleoprotein complex (the Lsr2 focus splits into two foci). We speculate that the Lsr2-mediated dynamic reorganization of DNA both behind and ahead of the moving replication fork may affect the dynamics of replisomes. In fact, we observed that the replisomes in WT cells frequently divided and merged, and that this was in contrast to the sparse splitting of replisomes in the Δ*lsr2* cells (Fig. 3A,C). Meanwhile, the Δ*lsr2* cells might have difficulty in organizing sibling chromosomes; the absence of Lsr2 presumably causes local chromosome disorganization, which could be the reason for the extend the B + D periods (Fig. 3B) and delay chromosome initiation in the daughter cells.

Given that the localization of the major Lsr2 focus on the chromosome is highly dynamic (Fig. 4, Video S1), we speculate that Lsr2 nucleoprotein complexes need to be dynamically assembled and disassembled during the course of chromosome replication, to organize the newly replicated DNA and to enable efficient progression of the DNA replication machinery, respectively. Notably, ChIP-on-chip experiments^19^, revealed that the few hundred Lsr2 binding sites are evenly distributed along the *M. smegmatis* chromosome (this analysis was carried out on non-synchronized cells). We speculate that the Lsr2 protein, during the course of DNA replication, bridges and organizes spatially distant regions of the chromosome. Thus, further comparison of Hi-C contact maps of the Δ*lsr2* and the wild type strains made on synchronized cells should provide an insight into the mechanism of Lsr2 binding sites selection (and their spatial organization) during the formation of highly dynamic nucleoprotein complexes in the course of DNA replication.

Previous in vitro studies showed that the N-terminal domain of Lsr2 is involved in oligomerization^5, 7^, enabling Lsr2 to form large nucleoprotein complexes by bridging distant DNA fragments. To elucidate the in vivo role of the N-terminal domain (NTD) of Lsr2, we constructed fluorescent reporter strains that produced a truncated form of Lsr2 lacking the NTD, Lsr2_ΔNTD_-Dendra2, or the wild-type protein, Lsr2-Dendra2 (Fig. 5B, Fig. S4), and compared the subcellular localizations of the fusion proteins using TLFM and high-resolution PALM (photoactivated localization microscopy).

Our TLFM analysis showed that Lsr2_ΔNTD_-Dendra2 was visible as diffuse fluorescence along the cell (Fig. 5A,C), whereas Lsr2-Dendra2 exhibited the foci described above. Notably, we did not observe any individual Lsr2_ΔNTD_-Dendra2 focus at any point during the cell cycle. In addition, the PALM analysis revealed that the ratio of diffusing versus immobile particles was considerably higher for the truncated form than for the wild-type protein (Fig. 5D), suggesting that a substantial fraction of the Lsr2_ΔNTD_-Dendra2 particles may be present in the cytoplasm rather than bound to the nucleoid. We note, however, that the high mobility of the Lsr2_ΔNTD_-Dendra2 particles may be the result of unstable (and transient) DNA binding and competition with other DNA binders, including NAPs. Thus, our in vivo observations are consistent with the previous in vitro experiments and support the hypothesis that the N terminus of the Lsr2 protein is indispensable for the formation of the massive nucleoprotein complexes that are visible as bright fluorescent foci in vivo.

We cannot exclude the possibility that the dynamic spatial organization of Lsr2 nucleoprotein complexes may partially depend on the formation of membrane-free microcompartments created as a result of crowding-induced liquid–liquid phase separation (LLPS). Recently, it has been demonstrated that LLPS may modulate assembly and localization of bacterial molecular macrocomplexes^32, 33^.

In sum, our results indicate that Lsr2 exerts pleiotropic effects in *M. smegmatis* including changes in the morphology of cell, the local chromosome structure, and the dynamics of chromosome replication. Some of these changes (e.g., alteration of the cell morphology) are presumably associated with transcriptional activity of Lsr2. In vivo, Lsr2 forms a nucleoprotein complex that exhibits a highly dynamic localization during the cell cycle. Moreover, the Lsr2 N-terminal domain, which is involved in oligomerization, is indispensable for the in vivo formation of nucleoprotein complexes. Given that the Lsr2 protein is essential during the infection of *M. tuberculosis*^15, 34, 35^ and exhibits pleiotropic activities including being a transcription factor^36^, Lsr2 appears to be an attractive target for the development of new antimicrobials.

## Materials and methods

### DNA manipulations, bacterial strains, and culture conditions

All plasmids used for transformation of *M. smegmatis* mc^2^ 155 were propagated in the *E. coli* DH5α strain. *E. coli* was grown in LB broth or on LB agar plates (Difco) supplemented with the proper antibiotic(s) and/or other compounds (100 µg/ml ampicillin, 50 µg/ml kanamycin, 50 µg/ml hygromycin, 0.004% X-Gal [5-bromo-4-chloro-3-indolyl-α-d-galactopyranoside]), accordingly to standard procedures^37^. *M. smegmatis* strains were grown in 7H9 broth supplemented with 10% OADC (oleic acid-albumin-dextrose-catalase; BD) and 0.05% Tween 80, or on 7H10 agar plates (Difco) supplemented with 10% OADC, 0.5% glycerol, 0.004% X-Gal, 2% sucrose and/or kanamycin or 50 µg/ml hygromycin. DNA manipulations were carried out using standard protocols^37^. Reagents and enzymes were obtained from Thermo Fisher, Roth, and Merck (Sigma-Aldrich). Oligonucleotides were synthesized by Genomed (Poland) or Merck (Sigma-Aldrich), and sequencing was performed by Genomed or Microsynth (Germany). To determine the growth curve for *M. smegmatis* strains in optimal conditions and during exposure to vancomycin, cells were grown at 37˚C in a final volume of 300 μl 7H9 (supplemented with OADC and Tween80), and optical density measurements were taken at 10-min intervals for 24–72 h using a Bioscreen C instrument (Growth Curves US). Bacterial strains, plasmids, and oligonucleotides are listed in Tables S1–S3 in the supplemental material. The construction of the *M. smegmatis* mc^2^ 155 mutant strains is detailed in Supplementary Information.

### Microscopy and cell staining

Snapshot imaging of *M. smegmatis* was performed using cells cultured to log phase (optical density at 600 nm [OD_600_] ~ 0.6). Cells were directly plated on an agar pad containing FM5-95 (0.5 µg/l; Thermo Fisher) for membrane staining or incubated with SYTO^TM^61 (1.6 µM) for 15 min prior to being plated on the agar pad for nucleoid staining. Real-time analyses were performed on solid medium in ibidi micro dishes and in liquid medium using a CellASIC ONIX platform and compatible B04A plates (Merck), as described previously^12, 13, 38^. In both cases, early log phase (OD_600_ ~ 0.2 to 0.4) *M. smegmatis* cultures grown in liquid medium were used. For membrane staining, cells were plated on an ibidi µ-dish (35 mm, low) with solid medium (7H10 supplemented with OADC) containing FM5-95 (0.5 µg/l). For NADA (fluorescent d-amino acid; Torcis Bio-Techne)/TMR-Tre (fluorescent trehalose; Torcis Bio-Techne) staining^39, 40^, cells loaded into the observation chamber were cultured in 7H9 (supplemented with OADC and Tween80). They were then subjected to 2-min pulse flows of 7H9-OADC-Tween 80 medium supplemented with NADA (0.5 mM) or TMR-Tre (100 µM) every hour. For Vancomycin-BODIPY (BODIPY™ FL Vancomycin; Thermo Fisher) staining, cells loaded into the observation chamber were exposed to fresh 7H9-OADC-Tween 80 for 5 h, followed by 7H9-OADC-Tween 80 supplemented with 0.5 µg/ml Vancomycin-BODIPY for 5 h, and then 7H9-OADC-Tween 80 supplemented with 1 µg/ml Vancomycin-BODIPY for 5 h (Fig. 3). All microfluidic experiments were performed under constant pressure (1.5 psi). Images were recorded at 10 min intervals using a Delta Vision Elite inverted microscope equipped with a 100 × oil immersion objective and an environmental chamber set to 37 °C.

Pictures were analyzed using the Fiji and R software packages (R Foundation for Statistical Computing, Austria; http://www.r-project.org), including the ggplot2 package^41^. The Shapiro–Wilk test was used to check whether the analyzed data have a Gaussian distribution and then for all measurements, a two-sided parametric Student’s t test was applied. To avoid the generation of false assumptions in the case of non-normal distributions, the statistical significance of differences in the measured values was confirmed with the nonparametric two-sided Wilcoxon test with minimum confidence intervals of 0.995.

### Atomic force microscopy (AFM)

To estimate Young’s modulus, *M. smegmatis* cultures were grown in 7H9-OADC-Tween 80 medium. Two milliliters of bacterial culture were centrifuged (6000 rpm for 5 min), washed twice with 7H9-OADC (to remove the Tween 80) and resuspended in 50 µl 7H9-OADC. Microscope slides were prepared as previously described^42^ by mixing polydimethylsiloxane (PDMS; Sylgard 184; Dow Corning) at a ratio of 15:1 (elastomer:curing agent). Air bubbles were removed from the mixture under negative pressure for 20 min. The PDMS mixture was dropped onto the microscope slides (VWR), which were spin-coated and baked at 80 °C for 10 min before use. A concentrated bacterial pellet was deposited on the surface of a PDMS-coated slide and incubated for 10 min. Then, the PDMS-coated slide was washed with 7H9-OADC to remove non-immobilized cells. AFM measurements were conducted in 7H9-OADC medium. The Young’s modulus was estimated using the PeakForce QNM scanning mode (scan rate, 1 Hz; amplitude, 100 nm) with PFQNM-LC-A-CAL cantilevers (resonant frequency, 45 kHz; spring constant, 0.1 N/m; Bruker). Young’s modulus was measured at five points of each cell using the NanoScope Analysis 1.9 software, and then the mean modulus value was calculated for *M. smegmatis* WT and Δ*lsr2* cells.

### Single-particle tracking

Lsr2-Dendra2 and Lsr2_ΔNTD_-Dendra2 cells were cultured to mid-log phase in rich medium (7H9 supplemented with 10% ADC and 0.05% Tween 80). Immediately before imaging, cells were spread onto agar pads (1% agarose in 7H9 poured into 1.0 × 1.0-cm GeneFrames; Thermo Fisher Scientific) and covered with a clean 22 × 22 0.17-mm coverslip. Imaging was carried out using a Zeiss Elyra P.1 inverted microscope equipped with an Andor iXon DU 897 Electron Multiplying Charge Coupled Device (EMCCD) camera and an alpha Plan-Apochromat 100×/1.46 Oil DIC M27 objective in combination with a 1.6 × Optovar (laser lines HR diode 50 mW 405 nm and HR DPSS 200mW 561 nm). The Z-axis was stabilized via the “definite focus” system. The samples were prebleached and the images were recorded using a 10.3-ms exposure per frame (561-nm laser, 30% intensity, 10 k frames in total) and increasing 405-nm excitation (continuous increase from 0.002 to 1%). All system was performed with an EMCCD gain of 250 and a Long Pass (LP) 570 filter set. Data were analyzed using Fiji^43^, Oufti^44^, and SMtracker^45^ software packages.

## Supplementary Information

Supplementary Video 1.

Supplementary Information 1.
