# Supplementary material for: Lsr2, a nucleoid-associated protein influencing mycobacterial cell cycle
Source: Sci Rep. 2021 Feb 3;11:2910. doi: 10.1038/s41598-021-82295-0 (PMC7858621; doi:10.1038/s41598-021-82295-0)
Supplement: Supplementary file 2 — Supplementary Information 1. [file 41598_2021_82295_MOESM2_ESM.docx]

**Supplementary Information**

**Lsr2, a peculiar nucleoid-associated protein influencing mycobacterial cell cycle**

Marta Kołodziej^1^, Damian Trojanowski^1^, Katarzyna Bury^2^, Joanna Hołówka^1^, Weronika Matysik^1^, Hanna Kąkolewska^1^, Helge Feddersen^3^, Giacomo Giacomelli^3^, Igor Konieczny^2^, Marc Bramkamp^3^, Jolanta Zakrzewska-Czerwińska^1*^

^1^ Department of Molecular Microbiology, Faculty of Biotechnology, University of Wrocław, Wrocław, Poland

^2^ Intercollegiate Faculty of Biotechnology, University of Gdansk and Medical University of Gdansk, Gdansk, Poland

^3^Christian-Albrechts-Universität zu Kiel, Institut für allgemeine Mikrobiologie, 24118 Kiel, Germany

^*^ Correspondence to: jolanta.zakrzewska-czerwinska@uwr.edu.pl

**Supplemental Video 1 (Video S1)**

Subcellular localization of Lsr2-FP during the cell cycle concerning the position of chromosome (HupB-FP), replisome (DnaN-FP) and segrosome (ParB-FP). TLM movies of representative cells of the Lsr2-mTurquoise2 (top-left panel), Lsr2-mCherry_HupB-EGFP (top-right panel) Lsr2-mTurquoise2_DnaN-mCherry (bottom-left panel) and the Lsr2-mCherry_ParB-mNeon (bottom-right panel) strains. After 10 hours (600 min) of growth under optimal conditions. Images were acquired every 10-min. Scale bar 5 µm.

**Supplemental Figures**

**
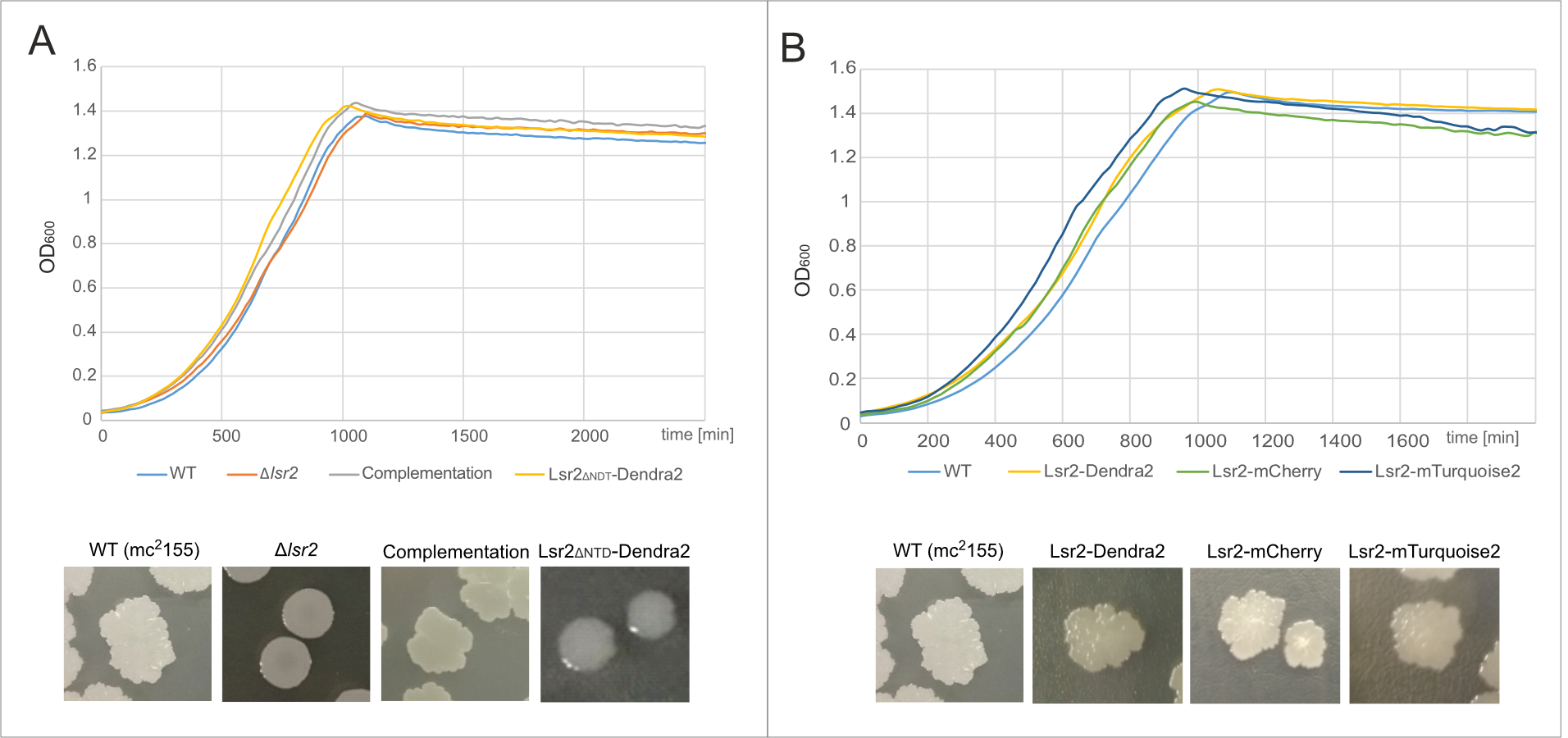
**

**Fig. S1. Phenotypic analysis of the *M. smegmatis* strains.**

Growth curve and colony morphology: **(A)** the wild-type (WT), Δ*lsr2*, Δ*lsr2*_p_NAT_*lsr2* (complementation), Lsr2_ΔNTD_**-**Dendra2 and **(B)** wild-type (WT), Lsr2-Dendra2, Lsr2-mCherry Lsr2-mTurquoise2.


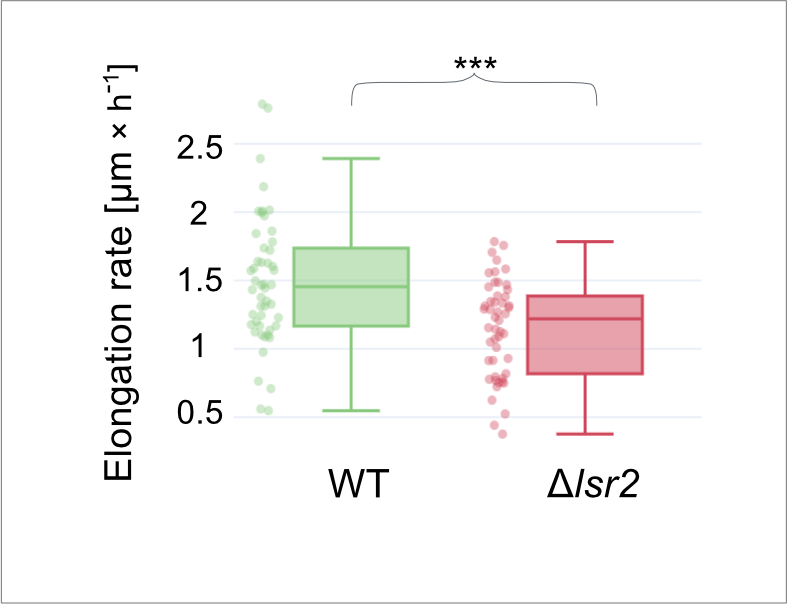


**Fig. S2 Analysis of the wild-type (WT), Δ*lsr2* elongation rate**

Box-plot comparison of the elongation rate of WT versus Δ*lsr2* cells (n = 50, statistical significance was defined as ***p < 0.0005, as assessed with a parametric double-sided t-test with pooled SD). The increase of cell length per hour [µm × h^-1^] was calculated by comparing the cell length measured shortly after cell division and at 50 min thereafter.


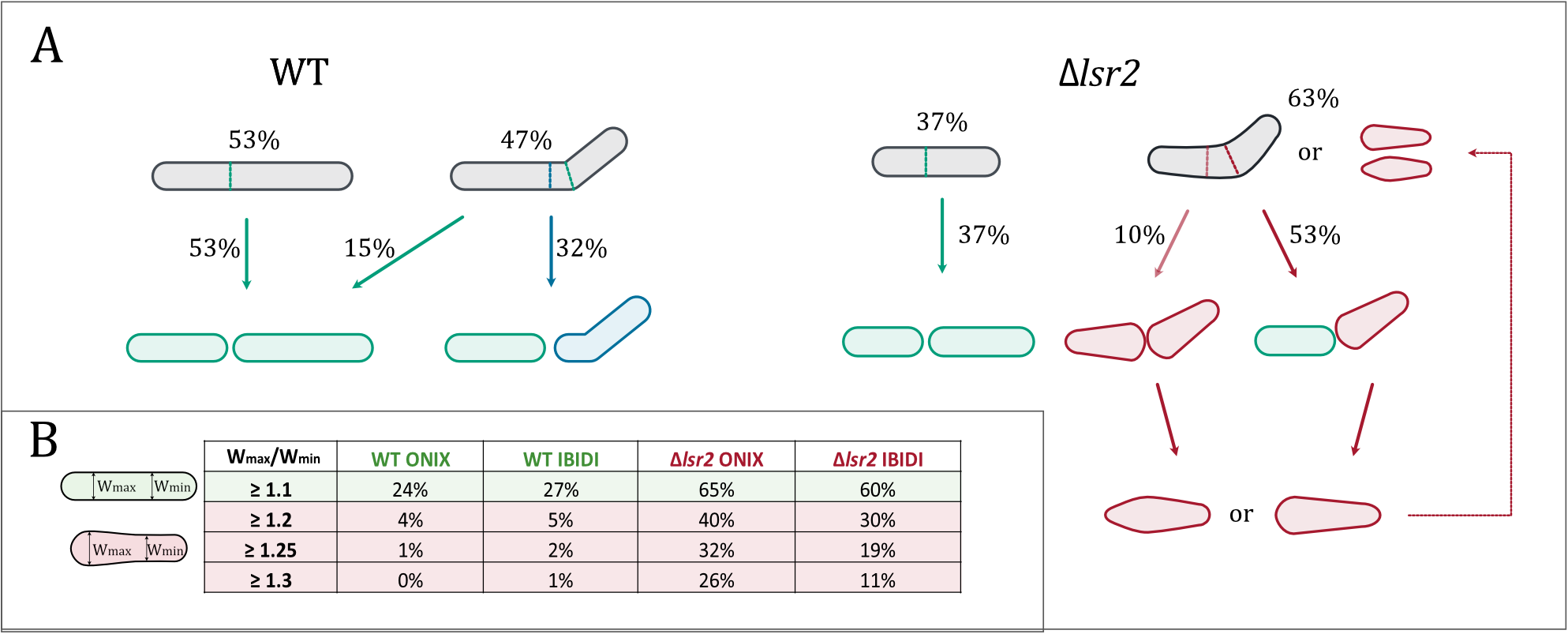


**Fig. S3. The formation of club-shaped Δ*lsr2* cells.**

**(A)** Schematic depiction of the cell division and formation of club-shaped cells in WT cells (n = 131) and Δ*lsr2* cells (n = 116). **(B)** Fraction of irregular-shaped cells (Wmax /Wmin >1) for each observation method.


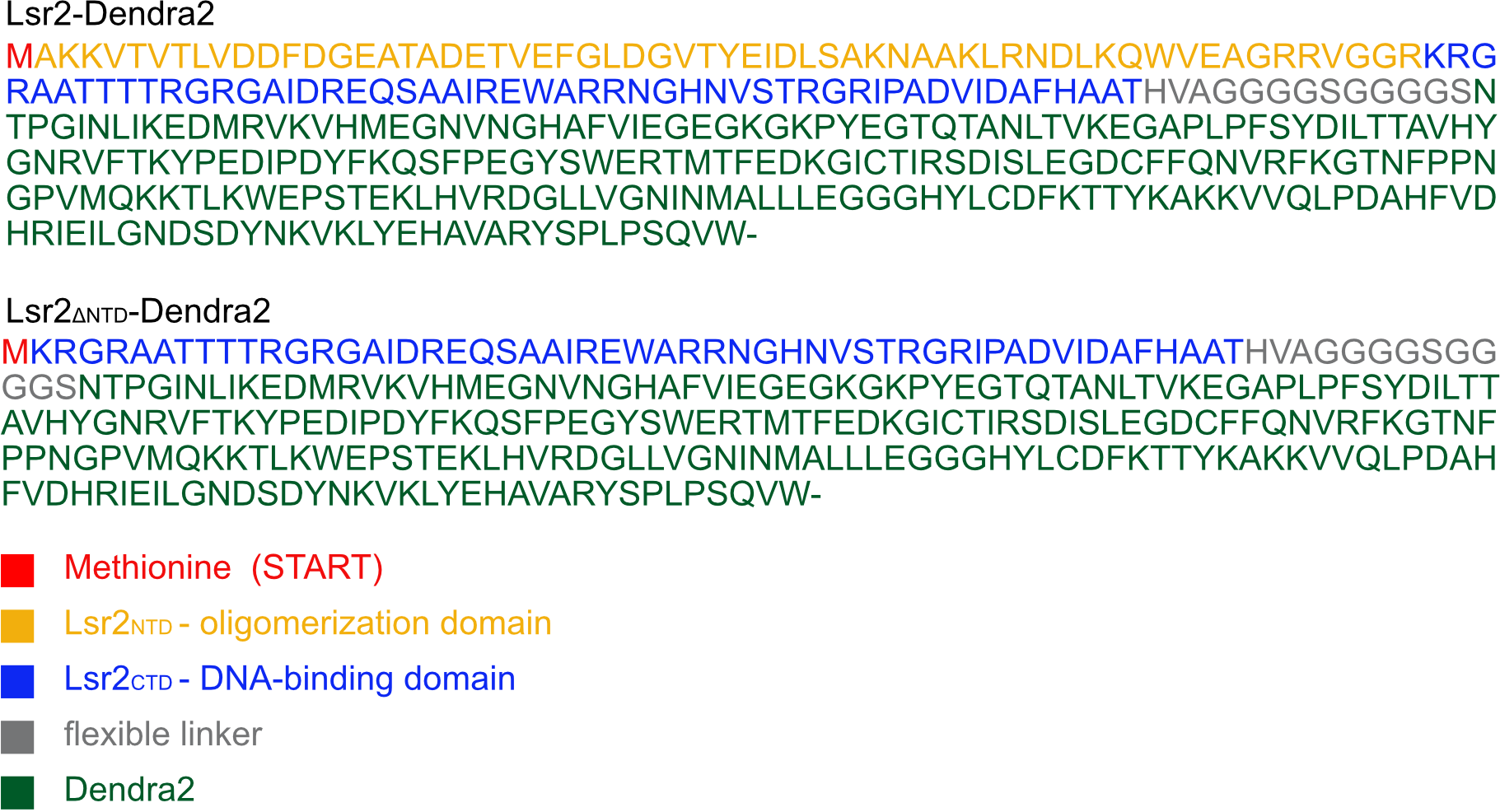


**Fig. S4. The amino acid sequence of Lsr2-Dendra2 fusion protein and truncated version Lsr2_ΔNTD_-Dendra2 (lacking the oligomerization domain [1]).**

**Supplemental Experimental Procedures**

***M. smegmatis* mc^2^ 155 mutant strains construction**

The allelic replacement of gene encoding Lsr2 (MSMEG_6092) protein with *lsr2-egfp*, *lsr2-mCherry*, *lsr2-mTurquoise2*, *lsr2-dendra2* or *lsr2_BD_-dendra2* was performed accordingly to Parish and Stoker [2]. The allelic replacement of gene encoding HupB (*MSMEG_2389*) with *hupB-egfp*, DnaN (*MSMEG_0001*) with *dnaN-mCherry* and ParB (*MSMEG_6938*) with *parB-mNeonGreen* was described previously [3–5]. In the case of Lsr2 fluorescence fusion, flanking sequences containing the *MSMEG_6091* and *MSMEG_6092-4* genes were amplified using as a template *M. smegmatis* mc^2^ 155 (WT) chromosomal DNA and primer pairs: Ms_6091_BamH_Fw/Ms_6091_Pac_Rv and Ms_6094_Hind_Fw/Ms_6092_Pml_Rv (Table S3). PCR products were cloned (by traditional restriction enzyme cloning protocol) into a p2NIL (kan*^R^*) vector to create a basic vector, prepared to clone fusion genes (p2NIL+flanks). The *egfp/mCherry/mTurquoise2/dendra2* gene with a short, flexible linker [6] encoding 10 amino acid sequence (GGGGSGGGGS) at the 5ʹ-terminus was PCR-amplified using primers listed in Tab. S3, and cloned to p2NIL+flanks vector using SLIC (Sequence and Ligation Independent Cloning) [7]. In the case of *lsr2_BD_-dendra2,* insert sequence was amplified using primer pairs Sma_START_lsr2BD_SLIC_Fw/ Dendra_SLIC_Rv and p2NIL-Lsr2-Dendra2 as a template, and cloned to p2NIL+flanks vector digested with SmaI and BamHI, instead of *lsr2* gene. For construction of *M. smegmatis* mc^2^ 155 strain with deletion of *lsr2* gene cloning strategy was similar to creating p2NIL+flanks (see above) but flanking sequences containing the *lsr2* upstream region were amplified using primer pairs Ms_6094_Hind_Fw/Ms_delta_lsr2_BamH_Rv.

All p2NIL derivatives were verified by sequencing (by Genomed or Microsynth). At the end, the pGoal17 cassette was cloned into the PacI site of each p2NIL derivative. *M. smegmatis* cells were then transformed with 200-1000 ng of NaOH/EDTA-treated plasmid DNA and unmarked mutants were selected accordingly to the procedure described previously. DCO mutants were analyzed by PCR, DNA sequencing and/or Western blotting.

Because within *lsr2* gene was found another antisense ORF (MSMEG_6093) (see Fig S5), we constructed two complementation strains producing Lsr2 and MSMEG_6093. The function of MSMEG_6093 is unknown and *in silico* analysis did not give any information about any hypothetical MSMEG_6093 homologs. Nevertheless, we decided to find out if the complementation of MSMEG_6093 reinstates *lsr2* deletion phenotype. For construction of the complementation strain and strains producing Lsr2 proteins and MSMEG_6093, mycobacteriophage L5-based integration-proficient vector pMV306 (Kan^R^/Hyg^R^) was used [8]. The pMV-*lsr2*/pMV-*lsr2* vector was obtained by amplification of *lsr2/MSMEG_6093* gene with upstream region on a template of *M. smegmatis* mc^2^ 155 (WT) chromosomal DNA using primers Ms_6094Fw/Ms_6093Fw and Ms_6093Fw/Rv. PCR product was cloned into pMV306 vector and verified by sequencing. *M. smegmatis* electrocompetent cells were then transformed with pMV306 derivatives and recombinants were selected using kanamycin (pMV306-*lsr2*), or hygromycin (pMV306_hyg_-*MSMEG_6093*). Transformants were analyzed by PCR and sequencing. Analysis of growth rate (in oxygen depletion condition) and observation of colony morphology/biofilm formation revealed that complementation of *lsr2* gene reinstates *lsr2* deletion phenotype, in contrast to complementation of MSMEG_6093. It means that the observed Δ*lsr2* phenotype is the consequence of lacking Lsr2 protein, not the disruption of MSMEG_6093 gene.


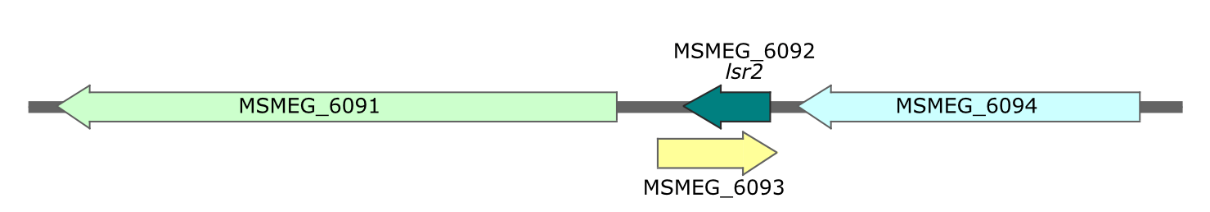


**Fig S5.** **Gene environment of *lsr2.*** *(https://mycobrowser.epfl.ch)* [7].

**Supplemental tables**

| **Table S1. Strains used in this study** | | | | |
| --- | --- | --- | --- | --- |
| **Name** | | **Relevant genotype** | **Source** | |
| WT | | *M. smegmatis* mc^2^ 155 | laboratory stock | |
| Δ*lsr2* | | *M. smegmatis* mc^2^ 155 Δ*lsr2* | this study | |
| Δ*lsr2*_p_NAT_*lsr2* | | *M. smegmatis* mc2 155 ∆*lsr2 attBL5::pMV306p_NAT_lsr2* | this study | |
| Δ*lsr2*_HupB-EGFP | | *M. smegmatis* mc^2^ 155 Δ*lsr2*_ *hupB::hupB-egfp* | this study | |
| HupB-EGFP | | *M. smegmatis* mc^2^ 155 *hupB::hupB-egfp* | [3] | |
| Δ*lsr2*_DnaN-mCherry | | *M. smegmatis* mc^2^ 155 *Δlsr2_dnaN::dnaN-mCherry* | this study | |
| DnaN-mCherry | | *M. smegmatis* mc^2^ 155 *dnaN::dnaN-mCherry* | [4] | |
| Lsr2-mCherry | | *M. smegmatis* mc^2^ *lsr2::lsr2-mCherry* | this study | |
| Lsr2-mTurquoise2 | | *M. smegmatis* mc^2^ *lsr2::lsr2-mTurquoise2* | this study | |
| Lsr2-EGFP | | *M. smegmatis* mc^2^ *lsr2::lsr2-EGFP* | this study | |
| Lsr2-Dendra2 | | *M. smegmatis* mc^2^ *lsr2::lsr2-Dendra2* | this study | |
| Lsr2_ΔNTD_-Dendra2 | | *M. smegmatis* mc^2^ *lsr2::lsr2* _ΔNTD_*-Dendra2* | this study | |
| Lsr2-mCherry_HupB-EGFP | | *M. smegmatis* mc^2^ *lsr2::lsr2-mCherry*_*hupB::hupB-egfp* | this study | |
| Lsr2-mCherry_ ParB-mNeonGreen | | *M. smegmatis* mc^2^ *lsr2::lsr2-mCherry*_ *parB::parB-mNeonGreen* | this study | |
| **Table S2. Plasmids used in this study** | | | |  |
| **Name** | **Plasmid features** | | **Source** |  |
| p2NIL | kanamycin resistance, *oriE*, suicide vector for allelic replacement | | [1] |  |
| pGoal17 | ampicillin resistance, *oriE*, selective PacI cassette (Goal) with *lacZ*, *sacB* and *kanR* genes | | [1] |  |
| p2NIL-Lsr2-EGFP-Goal | kanamycin resistance, *oriE*, *lsr2-egfp* fusion gene, PacI cassette | | this study |  |
| p2NIL-Lsr2-mCherry-Goal | kanamycin resistance, *oriE*, *lsr2-mCherry* fusion gene, PacI cassette | | this study |  |
| p2NIL-Lsr2-mTurquoise2-Goal | kanamycin resistance, *oriE*, *lsr2-* *mTurquoise2* fusion gene, PacI cassette | | this study |  |
| p2NIL-Lsr2-Dendra2-Goal | kanamycin resistance, *oriE*, *lsr2-Dendra2* fusion gene, PacI cassette | | this study |  |
| p2NIL- Δ*lsr2-*Goal | kanamycin resistance, *oriE*, PacI cassette | | this study |  |
| p2NIL-Lsr2_ΔNTD_-Dendra2-Goal | kanamycin resistance, *oriE*, *lsr2_BD-_Dendra2* fusion gene, PacI cassette | | this study |  |
| pMV306 | kanamycin resistance*, oriE, oriM, attP, attB* integrative vector for mycobacterial transformation | | [2] |  |
| pMV306-*lsr2* | kanamycin resistance, *oriE, attP, lsr2* gene under natural promoter | | this study |  |

| **Table S3. Oligonucleotides used in this study** | | |
| --- | --- | --- |
| **Name** | **Sequence 5’ to 3’** | **General description** |
| Ms_6094_Hind_Fw | GGAAGCTTCGGCAAAGGCGCTGCGG | MSMEG_6094/MSMEG_6094-92 amplification (deletion/fusions); HindIII |
| Ms_6092_Pml_Rv | GGCACGTGAGTTGCCGCGTGGAATGC | MSMEG_6094-92 amplification (fusions); PmlI |
| Ms_delta_lsr2_BamH_Rv | GGGGATCCTTCTGCTAGAGCATTGGTGAC | MSMEG_6094 amplification (deletion) |
| Ms_6091_BamH_Fw | CCGGATCCCGGAAGGTTGCCAGCACG | MSMEG_6091 amplification (deletion/fusions); BamHI |
| Ms_6091_Pac_Rv | GGTTAATTAAATGGTGTGCTCGACGGTC | MSMEG_6094 amplification (deletion/fusions); PacI |
| Ms_6093_Fw | CCGGTACCTGGGCGGCCGCGCC | MSMEG_6093 amplification (complementation) |
| Ms_6093_Rv | CCGATATCTCACCAATGCTCTAGCAGA | MSMEG_6093 amplification (complementation) |
| GFP_BamH_Rv | CCGGATCCTTACTTGTACAGCTCGTCCATG | *egfp* with linker amplification |
| linker_GFP_Pml_Fw | GGCACGTGCTGCCGGGCCCGGAGCTG |  |
| mCherry_Pml_L_Fw | ACACGTGGGCGGCGGCGGCTCGGGCGGCGGCGGCTCGATGGTGAGCAAGGGC | Linker addition to *mCherry* |
| GFP_BamH_Rv | CCGGATCCTTACTTGTACAGCTCGTCCATG |  |
| L2_mCherry_Fw_SLIC | CATTCCACGCGGCAACTCACCACGTGGGCGGCGG | *mCherry* with linker amplification to SLIC reaction |
| mCherry_STOP_Rv_SLIC | TTCCGGGATCCGTCGACCACCCGGATCCTTACTTG TACAGCTCG |  |
| L2-mTurquoise2-Fw | GGCTCGGCGGGCTCGGCGGCGGGCTCGGGCGAGTTCATGGTCTCCAAGGGCGAGGAGC | Linker addition to *mTurquoise2* |
| mTurquoise2-Stop-Kpn-Rv | ATGGGTACCTCACTTGTACAGCTCG |  |
| lsr2-mTurquoise2-SLIC-Fw | CATTCCACGCGGCAACTCACGGCTCGGCGGGCT | *mTurquoise2* with linker amplification to SLIC reaction |
| lsr2-mTurquoise2-SLIC-Rv | TTCCGGGATCCGTCGACCACATGGGTACCTCACTTGTACAGCT |  |
| L_Dendra_Fw | GTAGCCGGCGGCGGCGGCTCGGGCGGCGGCGGCTCGAACACCCCGGGAATTAACCTG | Linker addition to *dendra2,*  *dendra2* amplification |
| Dendra_STOP_BamH_Rv | GGATCCTCACCACACCTGGCTGGGC |  |
| L_dendra_SLIC_Fw | CACGCGGCAACTCACGTAGCCGGCGGCGG | *dendra2* with linker amplification to SLIC reaction |
| Dendra_SLIC_Rv | GGATCCTCACCACACCTGGC | *dendra2* amplification |
| Sma_START_lsr2BD_SLIC_Fw | TAGCAGAAGGGTCGGTGCCCGGGA**ATG**AAACGCGGTCGCG | *lsr2*_ΔNTD_*-dendra2* amplification to SLIC reaction  bold font - added codon START |
| Dendra_SLIC_Rv | GGATCCTCACCACACCTGGC |  |

**Supplemental References**

1. Summers, E.L., Meindl, K., Usón, I., Mitra, A.K., Radjainia, M., Colangeli, R., Alland, D., and Arcus, V.L. (2012). The structure of the Oligomerization domain of Lsr2 from Mycobacterium tuberculosis reveals a mechanism for chromosome organization and protection. PLoS One *7*.

2. Parish, T., and Stoker, N.G. (2000). Use of flexible cassette method to generate a double unmarked Mycobacterium tuberculosis tlyA plcABC mutant by gene replacement. Microbiology *146*, 1969–1975.

3. Hołówka, J., Trojanowski, D., Ginda, K., Wojtaś, B., Gielniewski, B., Jakimowicz, D., and Zakrzewska-Czerwińska, J. (2017). HupB Is a Bacterial Nucleoid-Associated Protein with an Indispensable Eukaryotic-Like Tail. MBio *8*.

4. Trojanowski, D., Ginda, K., Pióro, M., Hołówka, J., Skut, P., Jakimowicz, D., and Zakrzewska-Czerwińska, J. (2015). Choreography of the mycobacterium replication machinery during the cell cycle. MBio *6*.

5. Trojanowski, D., Hołówka, J., Ginda, K., Jakimowicz, D., and Zakrzewska-Czerwińska, J. (2017). Multifork chromosome replication in slow-growing bacteria. Sci. Rep. *7*.

6. Chen, X., Zaro, J.L., and Shen, W.C. (2013). Fusion protein linkers: Property, design and functionality. Adv. Drug Deliv. Rev. *65*, 1357–1369.

7. Jeong, J.Y., Yim, H.S., Ryu, J.Y., Lee, H.S., Lee, J.H., Seen, D.S., and Kang, S.G. (2012). One-step sequence-and ligation-independent cloning as a rapid and versatile cloning method for functional genomics Studies. Appl. Environ. Microbiol. *78*, 5440–5443.

8. Triccas, J.A., Parish, T., Britton, W.J., and Gicquel, B. (1998). An inducible expression system permitting the efficient purification of a recombinant antigen from *Mycobacterium smegmatis*. FEMS Microbiol. Lett. *167*, 151–156. Available at: https://academic.oup.com/femsle/article-lookup/doi/10.1111/j.1574-6968.1998.tb13221.x [Accessed March 26, 2020].
